# LINC00511 promotes cervical cancer progression by regulating the miR-497-5p/MAPK1 axis

**DOI:** 10.1007/s10495-022-01768-3

**Published:** 2022-09-14

**Authors:** Mingming Lu, Qing Gao, Yafei Wang, Jie Ren, Tingting Zhang

**Affiliations:** 1 Department of Gynecology, The Second Affiliated Hospital of Xi’an Jiaotong University, 710004 Shaanxi, China; 2 Department of Obstetrics and Gynecology, Medical Colleage, Guizhou Medical University, 550004 Guiyang, Guizhou, China; 3 Department of Gynecology, The Second Affiliated Hospital of Xi’an Jiaotong University, No. 157, Xiwu Road, Xi’an, China

**Keywords:** Cervical cancer, LINC00511, miR-497-5p, Mitogen-activated protein kinase 1

## Abstract

**Background:**

Long non-coding RNA (lncRNA) exhibits a crucial role in multiple human malignancies. The expression of lncRNA LINC00511, reportedly, is aberrantly up-regulated in several types of tumors. Our research was aimed at deciphering the role and mechanism of LINC00511 in the progression of cervical cancer (CC).

**Method:**

Quantitative real-time polymerase chain reaction (qRT-PCR) was performed to quantify the expression levels of LINC00511, miR-497-5p and MAPK1 mRNA in CC tissues and cell lines. Cell counting kit-8 (CCK-8), 5-bromo-2’-deoxyuridine (BrdU) and Transwell assays were conducted for detecting the proliferation, migration and invasion of CC cells. Dual-luciferase reporter gene experiments were performed to verify the targeting relationships amongst LINC00511, miR-497-5p and MAPK1. Besides, MAPK1 expression in CC cells was detected via Western blot after LINC00511 and miR-497-5p were selectively regulated.

**Results:**

Up-regulation of LINC00511 expression in CC tissues and cell lines was observed, which was in association with tumor size, clinical stage and lymph node metastasis of the patients. LINC00511 overexpression facilitated the proliferation, migration and invasion of CC cells, while opposite effects were observed after knockdown of LINC00511. Mechanistically, LINC00511 was capable of targeting miR-497-5p and up-regulating MAPK1 expression.

**Conclusion:**

LINC00511/miR-497-5p/MAPK1 axis regulates CC progression.

## Introduction

Cervical cancer (CC) is the most common gynecological malignancies among women worldwide, which poses a serious threat to females’ health, with approximately 570,000 CC new cases diagnosed and more than 275,000 deaths reported annually [1–4]. It is imperative to further clarify the molecular mechanism underlying CC tumorigenesis, so as to improve the therapeutics of CC.

It is well documented that long non-coding RNAs (lncRNAs) are regulators of the malignant phenotypes of cancer cells [5–8]. For example, the significant augmentation of ZEB2-AS1 expression in non-small cell lung cancer is reported, and its high expression markedly promotes the migration, invasion and epithelial-mesenchymal transition (EMT) of tumor cells [9]. LncRNA MIR4435-2HG potentiates the migration and invasion of prostate cancer cells via up-regulation of TGF-β1 expression [10]. Moreover, LINC00511 exhibits cancer-promoting effects in multiple types of cancers. For example, LINC00511 facilitates the growth of breast cancer by accelerating the transition of G1/S phase and repressing apoptosis [11]. LINC00511, whose expression is also markedly up-regulated in CC tissues and cell lines, expedites the proliferation of CC cells and suppresses autophagy and apoptosis by modulating PLD1 [12]. Nevertheless, the expression characteristics, biological functions and regulatory mechanisms of LINC00511 in CC warrant further clarification.

MicroRNAs (miRNAs) are capable of binding to 3’ untranslated region (3’-UTR) of mRNA, further inducing target mRNA degradation or translational inhibition and hence regulating gene expression at the post-transcriptional level [13]. Previous studies report that miRNAs play crucial roles in cancer progression [14–18]. For example, miR-335 impedes the proliferation of lung cancer cells by targeting Tra2β [15]. Bone metastasis of prostate cancer cells is associated with the down-regulation of miR-141-3p expression [16]. In CC, miR-497-5p, a well-known tumor suppressor in cancer biology, induces cell cycle arrest and represses cancer cell proliferation by targeting CBX4 [17, 18]. Nonetheless, the molecular mechanism of miR-497-5p dysfunction in CC requires further delineation.

In the present research, the expression pattern, biological function, clinical implication and underlying mechanism of LINC00511 in CC were investigated. Functional experiments substantiated that LINC00511, whose expression was up-regulated in CC, promoted the proliferation, migration and invasion of CC cells. In terms of mechanism, LINC00511 was validated to accelerate the progression of CC by regulating miR-497-5p/mitogen-activated protein kinase 1 (MAPK1) axis.

## Materials and methods

### Tissue samples

CC tissues and para-cancerous tissues were collected from the Guizhou Medical University. Specimens were obtained with signed informed consent. This research, endorsed by the Ethics Review Board of the Guizhou Medical University, followed all principles of the Declaration of Helsinki.

### Cell cultivation and transfection

Non-malignant cervical epithelial cell line (End1/E6E7) and CC cell lines (SiHa, HeLa, C4-1 and HT-3) were purchased from American Type Culture Collection (ATCC, Manassas, VA, USA). The cells were cultivated in Dulbecco’s Modified Eagle’s Medium (DMEM, Hyclone, Logan, UT, USA) which was added with 10% fetal bovine serum (FBS, Invitrogen, Carlsbad, CA, USA), in an incubator with saturated humidity containing 5% CO_2_ at 37 °C. pcDNA empty vector (NC), pcDNA-LINC00511 (LINC00511), siRNA normal control (si-NC), siRNAs against LINC00511 (si-LINC00511), negative control of miR-497-5p mimic (miR-NC), negative control of miR-497-5p inhibitors (miR-in), miR-497-5p mimic and miR-497-5p inhibitors were purchased from GenePharma Co., Ltd. (Shanghai, China). Transfection was performed employing Lipofectamine™ 2000 reagent (Invitrogen, Carlsbad, CA, USA) according to the manufacturer’s protocol.

### Quantitative real-time polymerase chain reaction (qRT-PCR)

Total RNA was isolated from tissues and cells employing TRIzol reagent (Invitrogen, Carlsbad, CA, USA) according to the manufacturer’s protocol. cDNA synthesis was conducted by reverse transcription utilizing a PrimeScript ^TM^ RT reagent Kit with gRNA Eraser (Invitrogen, Carlsbad, CA, USA). qRT-PCR was conducted employing a SYBR Premix Ex Taq II kit (Takara Bio, Inc., Otsu, Japan). β-actin and U6 served as the endogenous controls, and the quantification of relative expression was conducted employing 2^−ΔΔCt^ method. The sequences of primers were shown as follows: LINC00511: forward: 5′-CGCAAGGACCCTCTGTTAGG-3′; reverse: 5′-GAAGGCGGATCGTCTCTCAG-3′. MAPK1: forward: 5′-TCTGCACCGTGACCTCAA-3′; reverse: 5′-GCCAGGCCAAAGTCACAG-3′. miR-497-5p: forward: 5ʹ-CCTTCAGCAGCACACTGTGG-3ʹ; reverse: 5ʹ-CAGTGCAGGGTCCGAGGTAT-3ʹ. U6: forward: 5′-CTCGCTTCGGCAGCACA-3′; reverse: 5′-TGGTGTCGTGGAGTCG-3′. β-actin: forward: 5′-TCACCAACTGGGACGACATG-3′; reverse: 5′-GTCACCGGAGTCCATCACGAT-3′.

### Cell counting kit-8 (CCK-8) assay

HT-3 and C4-1 cells (2 × 10^3^/well) were seeded into a 96-well plate and cultured overnight. Subsequently, the cells in each well were incubated with 10 µL of CCK-8 reagent (Dojindo, Kumamoto, Japan) for 2 h, and then a microplate reader was used to detect the optical density (OD) values of the cells at 450 nm wavelength. With the same method, the OD values were detected for three consecutive days.

### 5-bromo-2’-deoxyuridine (BrdU) assay

After CC cells were seeded in 96-well plates at a density of 2 × 10^3^ cells/well and cultured for 24 h, the BrdU kit (BD Pharmingen, San Diego, CA, USA) was added into each well (final concentration: 10 µM), and the culture was continued for 8 h. Subsequently, the medium was removed, and cells were fixed with 4% paraformaldehyde for 30 min and then incubated with anti-BrdU antibody (Beyotime, Shanghai, China) for 1 h at room temperature. After the cells were rinsed with phosphate buffer saline (PBS) for 3 times, Hoechest staining solution (Beyotime, Shanghai, China) was used for marking cell nuclei. Three fields were randomly selected under a fluorescence microscope. Cell proliferation rate = BrdU positive cells / Hoechst positive cells × 100%.

### Transwell assay

Cell suspensions (2 × 10^5^ cells/mL) of CC cells were prepared utilizing serum-free medium. A total of 0.2 mL cell suspension was added to the upper compartment of the Transwell system (Nest, Wuxi, China) while the lower compartment was filled with the medium containing 10% FBS. After the cells were placed in the incubator for 12 h, the migrated and invaded cells were fixed with 4% paraformaldehyde and then stained with 0.5% crystal violet solution. Under a microscope, five visual fields were selected in each group for counting the cell number. For the invasion assay, the bottom of the Transwell system was coated with a layer of Matrigel (30 µg/well; BD, San Jose, CA, USA) before the inoculation of the cells.

### Western blot

The extraction of total protein from cells was performed employing RIPA lysis buffer (Sigma-Aldrich, St. Louis, MO, USA). After the samples were mixed with 5×loading buffer and denatured in boiling water for 5 min, the separation of the proteins was completed via SDS-PAGE, followed by subsequent transfer of separated proteins onto polyvinylidene difluoride (PVDF) membranes (Beyotime, Shanghai, China). After that, the membranes were blocked in 5% skimmed milk for 2 h at room temperature and then incubated with primary antibodies against MAPK1 (1: 5000; ab257525; Abcam, Shanghai, China) and β-actin (1: 2000; ab5694; Abcam, Shanghai, China) overnight at 4 °C. Next, the membranes were incubated with horseradish peroxidase-labeled secondary antibodies (1:1000, Beyotime, Shanghai, China) at room temperature for 2 h. The development of protein bands was conducted employing an enhanced chemiluminescent kit (Biozym, Hessisch Oldendorf, Germany).

### Dual-luciferase reporter gene assay

The amplification of wild-type (WT) LINC00511 sequence and MAPK1 3’UTR sequence was completed by PCR, followed by the selective mutation of the sequences. Next, mutant (MUT) LINC00511 sequence, MUT MAPK1 sequence, WT LINC00511-WT sequence and WT MAPK1 sequence were inserted into pmirGLO luciferase reporter vectors (Promega, Madison, WI, USA). Subsequently, pmirGLO-LINC00511-MUT, pmirGLO-LINC00511-MUT, mirGLO-MAPK1-MUT and pmirGLO-MAPK1-WT were transfected into HT-3 and C4-1 cells, respectively, together with the transfection of miR-497-5p mimics or miR-NC. After 24 h, the Dual-Luciferase Reporter Assay System (Promega, Madison, WI, USA) with a GloMax 20/20 Luminometer (Promega, Madison, WI, USA) were used to detect the luciferase activity of the cells in each group in line with manufacturer’s protocol.

### Statistical analysis

All experiments were performed in triplicate, and all experimental data expressed by mean ± standard deviation were analyzed utilizing GraphPad Prism 8.0 (GraphPad Software Inc., La Jolla, CA, USA). Kaplan-Meier method with log-rank test was performed to evaluate the relationship between LINC00511 expression and progression-free survival (PFS) and overall survival (OS) of CC patients. The comparison of data was performed using student’s *t*-test (between two groups) and one-way ANOVA (among multiple groups), with *P* < 0.05 statistically indicating a significant difference.

## Results

### LINC00511 was highly expressed in CC tissues and cells

**Fig. 1:**
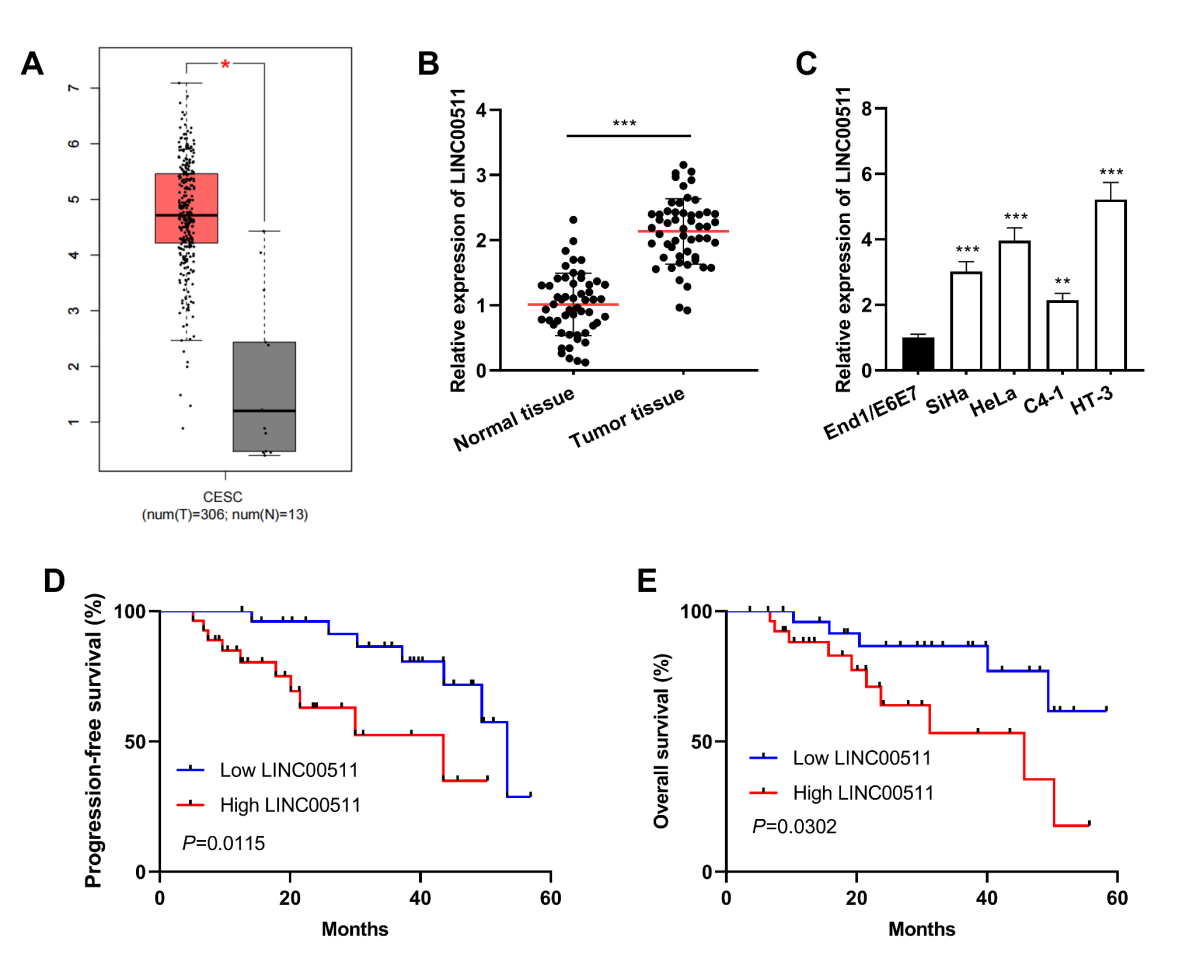
LINC00511 was highly expressed in CC tissues and cells. A. LINC00511 expression in cancer tissues and normal cervical tissues was analyzed utilizing GEPIA database. Red represents tumor tissues and grey represents normal tissues. CESC: cervical squamous cell carcinoma and endocervical adenocarcinoma, T (tumor): 306 cases, N (normal): 13 cases; |Log2FC| cutoff: 1; *p*-value cutoff: 0.01. LINC00511 expression in 53 pairs of CC tissues and adjacent tissues was quantified by qRT-PCR. qRT-PCR was employed to detect LINC00511 expression in normal cervical endometrial epithelial cells End1/E6E7 cells and CC cells (SiHa, HeLa, C4-1 and HT-3 cells). D-E. Kaplan-Meier curve was used to analyze the relationship between LINC00511 expression and progression-free survival and overall survival in CC patients. All experiments were performed in triplicate. *P < 0.05, **P < 0.01 and ***P < 0.001

First of all, the up-regulation of LINC00511 expression in CC samples was observed through the analysis of Gene Expression Profiling Interactive Analysis (GEPIA) database (gepia.cancer-pku.cn) (Fig. 1A). qRT-PCR was performed to quantify LINC00511 expression in cancer tissues and adjacent tissues collected from 53 CC patients, the results of which indicated a significant up-regulation of LINC00511 expression in CC tissue (Fig. 1B). Besides, qRT-PCR suggested that LINC00511 expression in CC cells (SiHa, HeLa, C4-1 and HT-3 cells) was remarkably higher than that in non-malignant cervical endometrial epithelial cell line End1/E6E7 (Fig. 1C). Additionally, to analyze the correlation between LINC00511 expression and clinicopathological features, 53 CC patients were divided into high expression group (n = 27) and low expression group (n = 26) with the median value of LINC00511 expression as the cutoff value. The results showed a remarkable correlation between LINC00511 expression and tumor size, clinical stage as well as lymph node metastasis, implying a cancer-promoting effect of LINC00511 in the development of CC (Table 1). In addition, we also analyzed the relationship between the expression of LINC00511 and the survival time of CC patients. Survival analysis showed that patients with high LINC00511 expression had significantly lower PFS and OS than the patients with low LINC00511 expression (Fig. 1D-E).

**Table 1:** Correlation between LINC00511 expression and multiple clinicopathological characteristics in CC patients. (n = 53)

Characteristics	Number	LINC00511 expression		*χ* ^*2*^	*P* value
		**High**	**Low**		
Age(years)					
< 50	25	15	10		
≥ 50	28	12	16	1.5531	0.2127
Gender					
Male	30	13	17		
Female	23	14	9	1.6020	0.2056
Tumor size (cm)					
≤ 5	22	6	16		
> 5	31	21	10	8.4328	0.0037
Clinical stage					
I~IIA	21	4	17		
IIB ~ IV	32	23	9	14.1588	0.0001
Distant metastasis					
Absent	28	14	14		
Present	25	13	12	0.021	0.884
Histological type					
Adenocarcinoma	15	7	8		
Squamous cell carcinoma	38	20	18	0.1531	0.6956
Lymph node metastasis					
Absent Present	29 24	8 19	21 5	13.9804	0.0001

### LINC00511 expedited proliferation, migration and invasion of CC cells

**Fig. 2:**
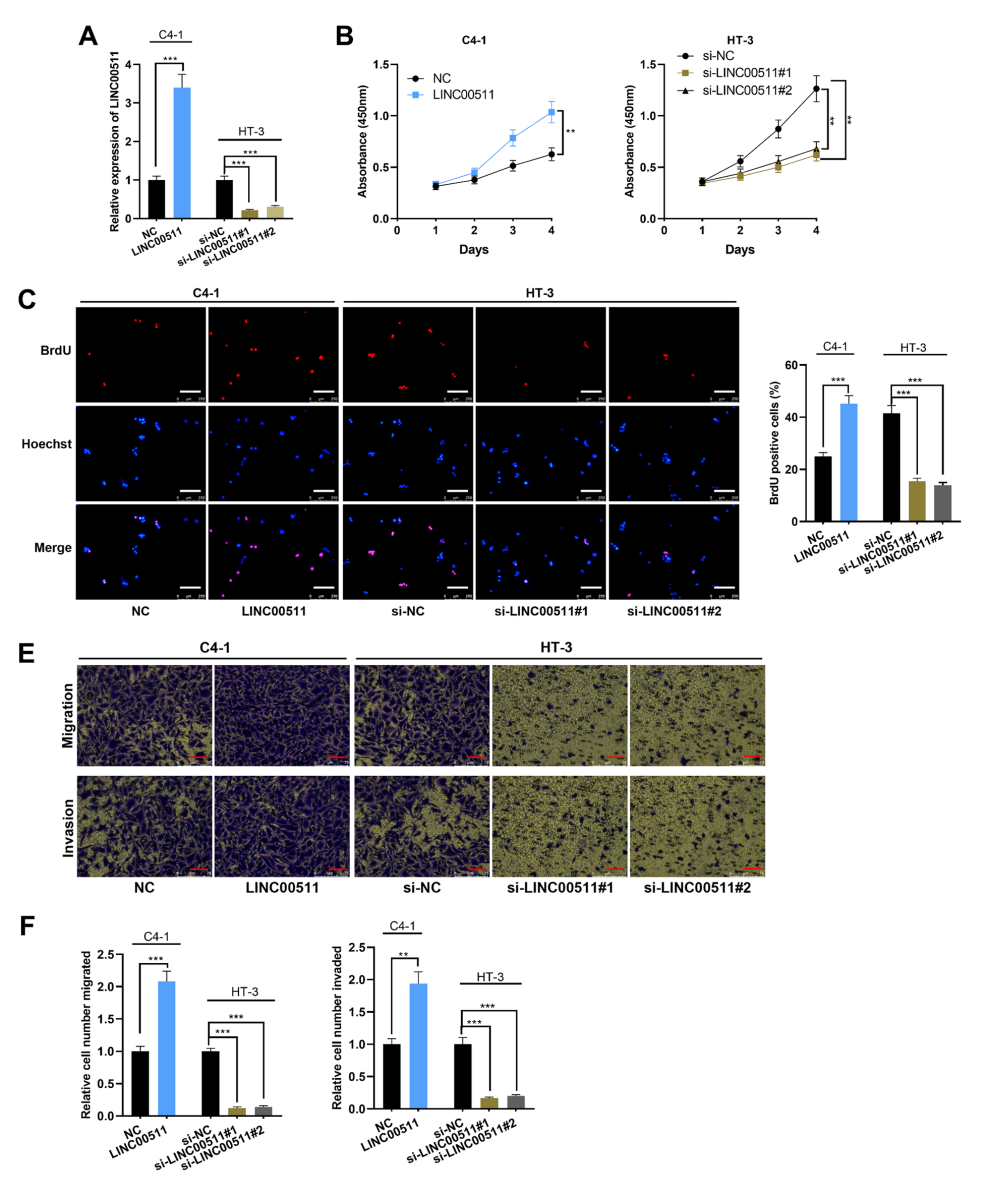
The effects of LINC00511 on proliferation, migration and invasion of CC cells. A. LINC00511 expression in C4-1 cells transfected with LINC00511 overexpression plasmid and HT-3 cells transfected with LINC00511 siRNAs was quantified employing qRT-PCR. B-D. CCK-8 method (B) and BrdU assay (C-D) were performed to detect the proliferation of CC cells after overexpression or knockdown of LINC00511. Scale bar, 250 μm. E-F. Transwell assay was utilized for monitoring migration and invasion of CC cells. Scale bar, 75 μm. All experiments were performed in triplicate. **P < 0.01 and ***P < 0.001

Among the CC cell lines, LINC00511 expression was the lowest in C4-1 cells and the highest in HT-3 cells (Fig. 1 C). Accordingly, LINC00511 overexpression plasmid and empty plasmid were transfected into C4-1 cells, si-LINC00511#1, si-LINC00511#2 and si-NC were transfected into HT-3 cells, and the gain-of-function and loss-of-function models were established (Fig. 2 A). Following that, CCK-8 and BrdU assays were implemented, which indicated overexpression of LINC00511 significantly promoted the proliferation of CC cells (Fig. 2B-D). Transwell experiments were also performed, and it was revealed that overexpression of LINC00511 promoted the metastatic potential of C4-1 cells (Fig. 2E-F). Conversely, compared with the control group, LINC00511 silencing with siRNAs inhibited the proliferation, migration and invasion of HT-3 cells (Fig. 2B-F). These data suggested that LINC00511 positively regulated the malignant phenotypes of CC cells.

### LINC00511 directly targeted miR-497-5p

**Fig. 3:**
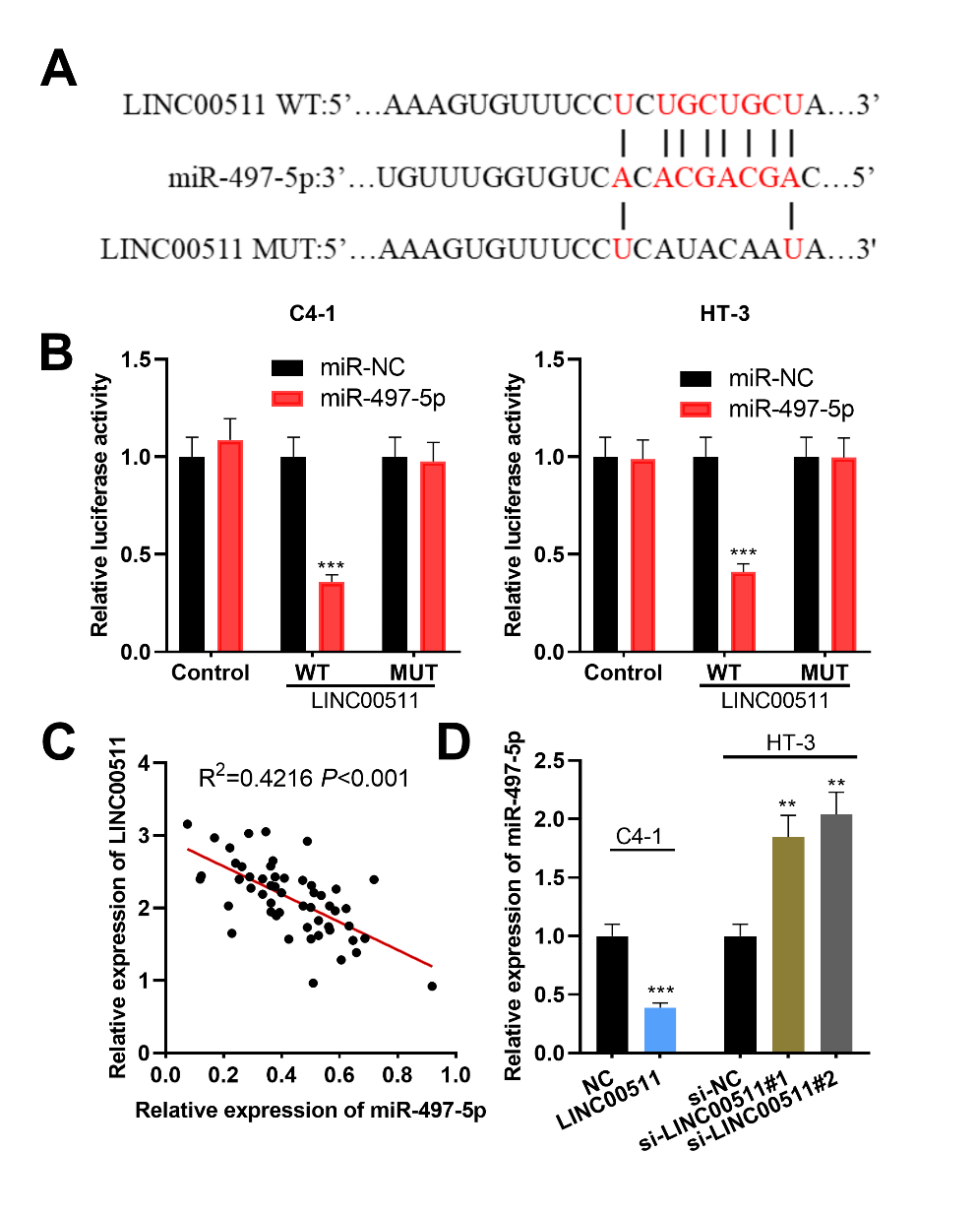
MiR-497-5p was the target of LINC00511 in CC cells. A. Bioinformatics was utilized for the prediction of binding site between LINC00511 and miR-497-5p. B. Dual-luciferase reporter gene experiment was employed for verifying the binding relationship between miR-497-5p and LINC00511. C. The correlation between LINC00511 and miR-497-5p expressions in CC tissues. D. MiR-497-5p expression in CC cells with LINC00511 overexpression or knockdown was assessed employing qRT-PCR. All experiments were performed in triplicate. **P < 0.01 and ***P < 0.001

Next, bioinformatics analysis with ENCORI database (http://starbase.sysu.edu.cn/) was utilized for predicting the downstream miRNAs of LINC00511. The results indicated a potential binding site between LINC00511 and miR-497-5p (Fig. 3 A). Subsequently, dual-luciferase reporter assay was conducted, and as shown, overexpression of miR-497-5p led to the reduction of the luciferase activity of wild-type LINC00511 reporter whereas miR-497-5p transfection did not affect the luciferase activity of mutant LINC00511 reporter (Fig. 3B). Additionally, the data from qRT-PCR assays indicated a negative correlation between miR-497-5p expression and LINC00511 expression in the CC samples (Fig. 3 C). Additionally, overexpression of LINC00511 remarkably impeded miR-497-5p expression in C4-1 cells while knockdown of LINC00511 markedly increased miR-497-5p expression in HT-3 cells (Fig. 3D). These results implied that miR-497-5p was a direct target of LINC00511 and its expression could be negatively regulated by LINC00511.

### MiR-497-5p exhibited a tumor-suppressive role in CC

**Fig. 4:**
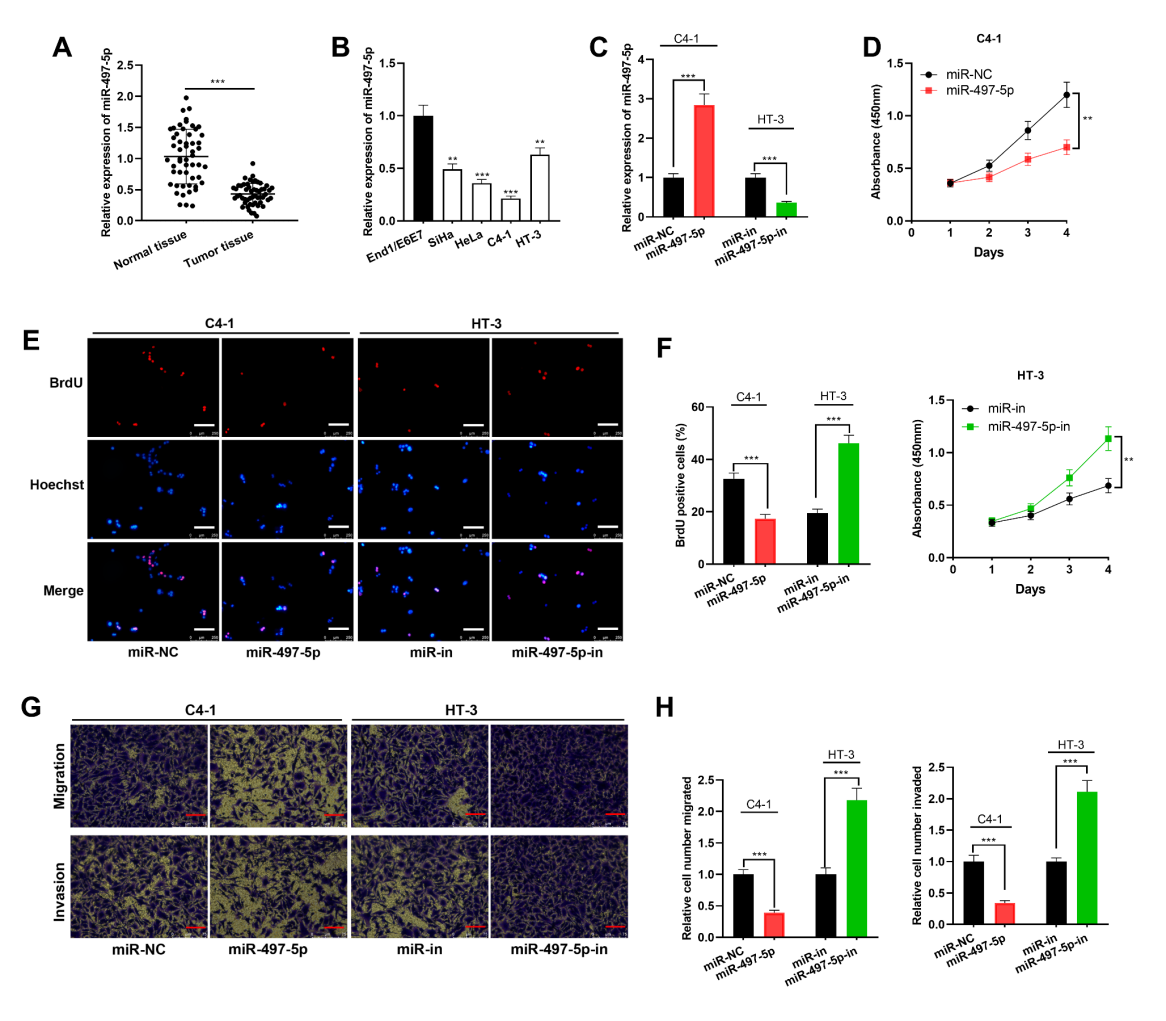
The effects of miR-497-5p on proliferation, migration and invasion of CC cells. A. MiR-497-5p expression in 53 pairs of CC tissues and adjacent tissues was quantified employing qRT-PCR. B. MiR-497-5p expression in End1/E6E7 cells and CC cell lines was detected employing qRT-PCR. C. qRT-PCR was utilized for quantifying miR-497-5p expression in CC cells transfected with miR-497-5p mimics or inhibitors. D-F. CCK-8 assay (D) and BrdU assay (E-F) were implemented for detecting the proliferation of CC cells. Scale bar, 250 μm. G-H. Transwell assay was utilized for monitoring the migration and invasion of CC cells. Scale bar, 75 μm. All experiments were performed in triplicate. **P < 0.01 and ***P < 0.001

For further clarifying the role of miR-497-5p in CC, expression characteristics of miR-497-5p in CC tissues and cell lines were detected by qRT-PCR, showing a significantly lower expression of miR-497-5p in CC tissues and cells than that in adjacent tissues and End1/E6E7 cell line (Fig. 4 A-B). Subsequently, the transfection of miR-497-5p mimics into C4-1 cells and the transfection of miR-497-5p inhibitors into HT-3 cells were performed (Fig. 4 C). Following that, CCK-8, BrdU and Transwell assay were implemented, and the results suggested that the up-regulation of miR-497-5p expression repressed the proliferation, migration and invasion of C4-1 cell, and the inhibition of miR-497-5p expression promoted these malignant phenotypes of HT-3 cells, suggesting that miR-497-5p was a tumor suppressor in CC (Fig. 4D-H).

### MiR-497-5p counteracted the biological effects of LINC00511 in CC

**Fig. 5:**
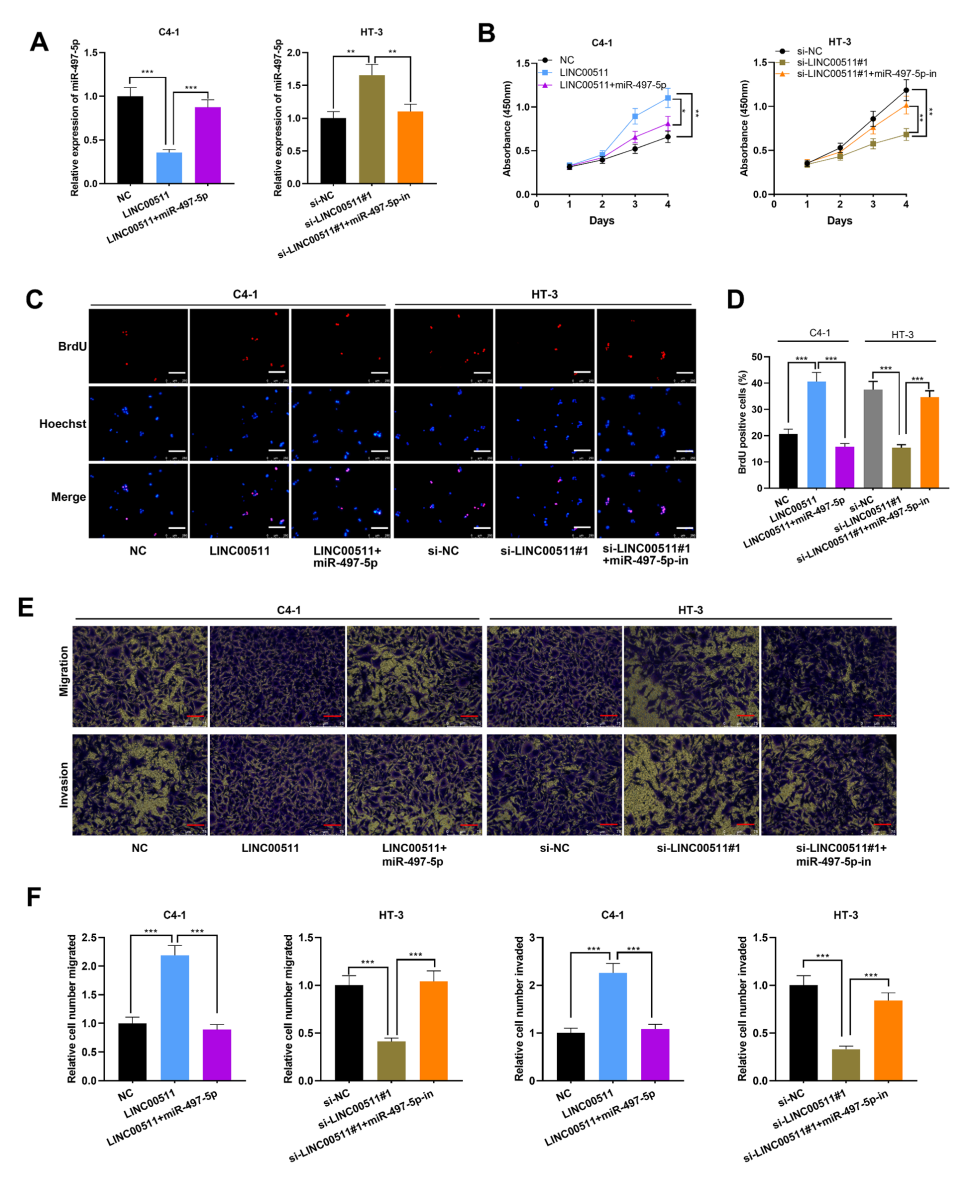
MiR-497-5p counteracted LINC00511’s effects on the proliferation, migration and invasion of CC cells. MiR-497-5p mimics and LINC00511 overexpression plasmids were co-transfected into C4-1 cells, and miR-497-5p inhibitors and LINC00511 siRNA (si-LINC00511#1) were co-transfected into HT-3 cells. The relative expression of miR-497-5p in C4-1 and HT-3 cells after transfection was detected by qRT-PCR. B-D. Cell proliferation was assessed utilizing CCK-8 assay (B) and BrdU assay (C-D). Scale bar, 250 μm. E-F. The migration and invasion of CC cells were monitored employing Transwell assay. Scale bar, 75 μm. All experiments were performed in triplicate. *P < 0.05, **P < 0.01 and ***P < 0.001

To further pinpoint the underlying mechanism and specific function of LINC00511/miR-497-5p axis in CC cells, miR-497-5p mimics and miR-497-5p inhibitors were co-transfected into C4-1 cells with LINC00511 overexpression and HT-3 cells with LINC00511 knockdown, respectively (Fig. 5 A). Functional experiments revealed that overexpression of miR-497-5p partially weakened the promotive effects of proliferation, migration and invasion of C4-1 cells ascribed to overexpression of LINC00511 while miR-497-5p inhibitors partially counteracted the inhibitory effects of knockdown of LINC00511 on proliferation, migration and invasion of HT-3 cells (Fig. 5B-F). These data revealed that LINC00511 regulated CC cell proliferation, migration and invasion by modulating miR-497-5p.

### LINC00511/miR-497-5p axis regulated MAPK1 expression

**Fig. 6:**
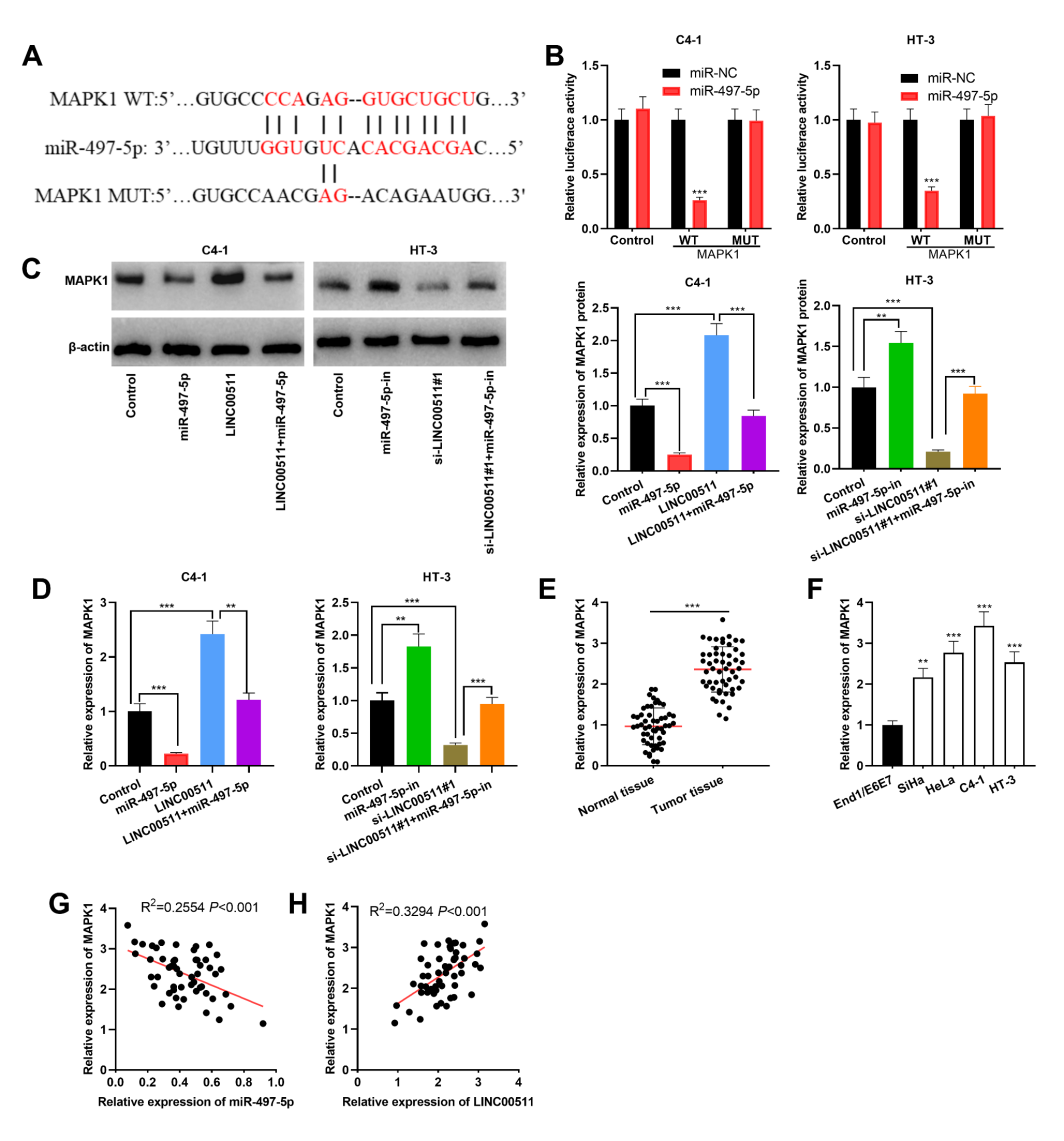
MAPK1 was the target of miR-497-5p in CC cells. A. Bioinformatics was utilized for the prediction of the binding site between miR-497-5p and 3’UTR of MAPK1. B. Dual-luciferase reporter gene experiment was implemented for verifying the targeted relationship between miR-497-5p and 3’UTR of MAPK1. C-D. Western blot (C) and qRT-PCR (D) were employed for quantifying MAPK1 expression in CC cells after transfection. E. MAPK1 mRNA expression in CC tissues and adjacent tissues of 53 patients was detected employing qRT-PCR. F. MAPK1 mRNA expression in CC cells and End1/E6E7 cells was detected utilizing qRT-PCR. G-H. The correlations between MAPK1 mRNA and miR-497-5p (G), and MAPK1 mRNA and LINC00511 (H) in CC tissues. All experiments were performed in triplicate. **P < 0.01 and ***P < 0.001

ENCORI database suggested a probable binding site between miR-497-5p and 3’UTR of MAPK1, which possessed cancer-promoting effects in CC [19] (Fig. 6 A). In dual-luciferase reporter assay, miR-497-5p mimics were observed to suppress the luciferase activity of wild type MAPK1 reporter whereas no significant effect of miR-497-5p on the luciferase activity of mutant MAPK1 reporter was observed (Fig. 6B). Furthermore, overexpression of miR-497-5p or knockdown of LINC00511 triggered a reduction of MAPK1 protein and mRNA expressions in C4-1 and HT-3 cells while the transfection of miR-497-5p inhibitors or LINC00511 overexpression plasmid promoted the expression of MAPK1 (Fig. 6 C-D). Meanwhile, miR-497-5p overexpression partially weakened the up-regulation of MAPK1 expression resulted from LINC00511 overexpression, while the inhibition of miR-497-5p expression partially counteracted the reduction of MAPK1 expression induced by LINC00511 knockdown (Fig. 6 C-D). Furthermore, qRT-PCR results showed an augmentation of MAPK1 mRNA expression in CC tissues and cells (Fig. 6E-F), and a negative correlation between miR-497-5p expression and MAPK1 mRNA expression and a positive correlation between LINC00511 expression and MAPK1 mRNA expression were found in CC tissues (Fig. 6E-H). These experimental findings suggested that MAPK1 was a downstream target of miR-497-5p, and LINC00511 could indirectly up-regulate MAPK1 expression by modulating miR-497-5p expression.

## Discussion

Currently, surgery, chemotherapy, radiotherapy, targeted therapy and immunotherapy are the main treatment strategies for cancers [20–22]. However, these methods still couldn’t cure most of the human malignancies, especially the metastatic diseases. In recent years, non-coding RNA therapeutics attracted a lot of attention, which are promising to improve the prognosis of the patients [22]. LncRNAs possess multiple functions including serving as a host gene of miRNA, preventing RNA and protein from binding to targets, or acting as a molecular scaffold to guide proteins to their targets [23]. LncRNAs exhibit a pivotal regulatory role in modulating the proliferation, migration and invasion of CC cells. For example, lnc-UICC is highly expressed in CC and accelerates the growth and metastasis of CC cells in vivo and in vitro [24]; the up-regulation of lncRNA HOXD-AS1 in CC regulates the proliferation of CC cells by activating Ras/ERK signaling pathway [25]. Reportedly, LINC00511 knockdown weakens the proliferation, migration and invasion of HeLa and C33A cells [26]. In another research, LINC00511 expression is increased in CC tissue, and LINC00511 overexpression significantly facilitates the proliferation and represses the apoptosis of HeLa cells [27]. In the current research, we verified that LINC00511 was highly expressed in CC tissues and cells, and its promoting effects on the proliferation, migration and invasion of CC cells were further validated, which was consistent with previous reports [12, 26, 27].

MiRNA exhibits a crucial role in CC progression. For instance, miR-218, whose expression is down-regulated in CC tissues and cells, represses the viability and induces the apoptosis of CC cells [28]; miR-501 expression is up-regulated in CC, and miR-501 expedites the proliferation, migration and invasion of CC cells by targeting CYLD [29]. Besides, miR-497-5p exhibits a cancer-inhibiting effect in lot of tumors. In hepatocellular carcinoma, the proliferation, migration and invasion of cancer cells were inhibited by miR-497-5p [30]; in non-small cell lung cancer, miR-497-5p suppresses the proliferation and invasion of cancer cells by targeting FGF2 [31]. In CC, miR-497-5p also functions as a tumor suppressor [18]. Herein, the inhibitory impacts of miR-497-5p on the malignant phenotypes of CC cells were further validated.

LncRNAs serve as competitive endogenous RNAs (ceRNA, or molecular sponge) for miRNAs, thereupon reducing the binding between miRNAs and mRNAs, and thereby modulating gene expression. LINC00511 functions as a molecular sponge in several types of cancers. For example, in gastric cancer, LINC00511, serving as the ceRNA of miR-124-3p, regulates PDK4 expression, hence promoting the growth of tumors [32]. Besides, it promotes the proliferation and metastasis of osteosarcoma cells by sponging miR-618 and up-regulating MAEL expression [33]. Herein, for the first time, our study reported that LINC00511 directly targeted miR-497-5p, and miR-497-5p partially counteracted the functions of LINC00511 on the proliferation, migration and invasion of CC cells. It was concluded that LINC00511 acted as a ceRNA for miR-497-5p, and our demonstrations provided a novel mechanism by which LINC00511 regulated CC progression.

Mitogen-activated protein kinase 1, encoded by human MAPK1 gene and termed as extracellular signal-regulated kinase (ERK), is a member of MAPK family, which is a crucial protein kinase in signal transduction, with serine/threonine kinase activity, and is indispensable for multiple biological processes [34–36]. For instance, the up-regulation of MAPK1 expression contributes to the augmentation of survivin expression, thereupon enhancing the resistance of multiple myeloma cells to vincristine [37]. Another study reports that inhibiting MAPK1 expression can attenuate YAP protein expression and then modulate Hippo pathway, thus constraining the migration and invasion of non-small cell lung cancer cells [38]. Importantly, MAPK1 is a cancer-promoting factor in CC. MAPK1 expression in CC tissues is significantly higher than that in adjacent tissues, and MAPK1 knockdown results in the down-regulation of MMP-2, MMP-9, Snail, TIMP-1 and TIMP-2 expressions, which remarkably suppresses the viability and invasion of HeLa cells [39]. In the current research, MAPK1 was confirmed to be the downstream target of miR-497-5p in CC cells, and LINC00511 could up-regulate MAPK1 expression through sponging miR-497-5p. Our data further clarified the regulatory mechanism of MAPK1 in CC.

In summary, our research reports that LINC00511 expression is up-regulated in CC and associated with adverse pathological characteristics of the patients, and LINC00511 promotes the malignant phenotypes of CC cells. In terms of mechanism, LINC00511 exhibits a cancer-promoting effect via the regulation of miR-497-5p/MAPK1 axis. Our work provides a novel ceRNA network involved in CC progression, and LINC00511 may be a therapeutic target for this disease. Notably, we observed that miR-497-5p restoration only partly counteracted the biological effects of LINC00511 on CC cells, ant this suggested that LINC00511 could probably modulate CC progression via other mechanisms. The other downstream targets / mechanisms of LINC00511 in CC remain to be deciphered in the following studies.

## Data Availability

The data used to support the findings of this study are available from the corresponding author upon request.

## References

[1] Wei 1 H, Qiu Y-Q, Zeng Q-S, Wang S-F, Yi C-J (2020 Apr) LncRNA UCA1 Regulates Proliferation, Migration and Invasion of Cervical Cancer Cells by Targeting miR-145. Eur Rev Med Pharmacol Sci 24(7):3555–3564 10.26355/eurrev_202004_20816 32329829

[2] Weizhi You S, Li R, Du J, Zheng A, Shen (2018 Jan) Epidemiological study of high-risk human papillomavirus infection in subjects with abnormal cytological findings in cervical cancer screening. Exp Ther Med 15(1):412–418 10.3892/etm.2017.5357 PMC5763653 29375696

[3] Kevin Y, Guo L, Han X, Li AV, Yang J, Lu S, Guan H, Li Y, Yu Y, Zhao J, Yang (2017) Hong Zhang. Novel Proteasome Inhibitor Delanzomib Sensitizes Cervical Cancer Cells to Doxorubicin-Induced Apoptosis via Stabilizing Tumor Suppressor Proteins in the p53 Pathway. Oncotarget. Dec 12;8(69):114123–114135 10.18632/oncotarget.23166 PMC5768391 29371974

[4] Cecilia Orbegoso K, Murali, Susana Banerjee (2018). The Current Status of Immunotherapy for Cervical Cancer. Rep Pract Oncol Radiother. Nov-Dec.

[5] Aalijahan H, Ghorbian S (2019). Long non-coding RNAs and cervical cancer. Exp Mol Pathol.

[6] Arun, Renganathan (2017). Emanuela Felley-Bosco. Long Noncoding RNAs in Cancer and Therapeutic Potential. Adv Exp Med Biol.

[7] Alessia Mongelli F, Martelli A, Farsetti, Carlo Gaetano (2019). The Dark That Matters: Long Non-coding RNAs as Master Regulators of Cellular Metabolism in Non-communicable Diseases. Front Physiol. May 22;10:369 10.3389/fphys.2019.00369 PMC6539782 31191327

[8] Pavan Kumar Puvvula. LncRNAs Regulatory Networks in Cellular Senescence.Int J Mol Sci. 2019 May28;20(11):2615 10.3390/ijms20112615 PMC6600251 31141943

[9] Xu H, Yu B, Shen W, Jin C, Wang L, Xi Y(2020) Over-expression of long non-coding RNA ZEB2-AS1 may predict poor prognosis and promote the migration, invasion, and epithelial-mesenchymal transition of tumor cells in non-small cell lung cancer.Int J Biol Markers. Jul 2;1724600820938385. 10.1177/1724600820938385 32611283

[10] Zhang H, Meng H, Huang X, Tong W, Liang X, Li J, Zhang C, Chen M (2019 Oct) lncRNA MIR4435-2HG Promotes Cancer Cell Migration and Invasion in Prostate Carcinoma by Upregulating TGF-β1. Oncol Lett 18(4):4016–4021 10.3892/ol.2019.10757 PMC6732992 31516603

[11] Zhang J, Sui S, Wu H, Zhang J, Zhang X, Xu S, Da Pang. The Transcriptional Landscape of lncRNAs Reveals the Oncogenic Function of LINC00511 in ER-negative Breast Cancer. Cell Death Dis. 2019 Aug 8;10(8):599 10.1038/s41419-019-1835-3 PMC6687715 31395854

[12] Yangyang Shi M, Liu Y, Huang J, Zhang L, Yin. Promotion of Cell Autophagy and Apoptosis in Cervical Cancer by Inhibition of Long Noncoding RNA LINC00511 via Transcription Factor RXRA-regulated PLD1.J Cell Physiol. 2020 Feb17;235(10):6592–6604 10.1002/jcp.29529 32067228

[13] Hou Z-LM, Li Y-L, Wang D-T, Yuan T-W, Wei J-L, Zhao B-T, Lou J-T, Zhao X-T, Jin Y (2015) You-Xin Jin. MicroRNA-34a Inhibits the Proliferation and Promotes the Apoptosis of Non-Small Cell Lung Cancer H1299 Cell Line by Targeting TGFβR2. Tumour Biol. Apr;36(4):2481-90 10.1007/s13277-014-2861-5 25501507

[14] Zhang X, Li Y, Qi P Zhongliang Ma. Biology of MiR-17-92 Cluster and Its Progress in Lung Cancer. Int J Med Sci. 2018 Sep 7;15(13):1443–1448 10.7150/ijms.27341 PMC6216058 30443163

[15] Liu J, Bian T, Feng J, Qian L, Zhang J, Jiang D, Zhang Q, Li X, Liu Y, Shi J (2018 Feb) miR-335 Inhibited Cell Proliferation of Lung Cancer Cells by Target Tra2β. Cancer Sci 109(2):289–296 10.1111/cas.13452 PMC5797811 29161765

[16] Shuai Huang Q, Wa J, Pan X, Peng D, Ren Y, Huang X, Chen Y, Tang. Downregulation of miR-141-3p Promotes Bone Metastasis via Activating NF-κB Signaling in Prostate Cancer.J Exp Clin Cancer Res. 2017 Dec4;36(1):173 10.1186/s13046-017-0645-7 PMC5716366 29202848

[17] Lan Feng K, Cheng R, Zang Q, Wang J, Wang (2019 Sep) miR-497-5p Inhibits Gastric Cancer Cell Proliferation and Growth Through Targeting PDK3. Biosci Rep 6(9):BSR20190654 10.1042/BSR20190654 PMC6732365 31409724

[18] Chen Y, Du J, Wang Yu, Shi H, Jiang Q, Wang Y, Zhang H, Wei Y, Xue W, Pu Z, Gao Y, Li D, Feng Y, Jing Yan, Jing Zhang. MicroRNA-497-5p Induces Cell Cycle Arrest Of Cervical Cancer Cells In S Phase By Targeting CBX4.Onco Targets Ther. 2019 Dec2;12:10535–10545 10.2147/OTT.S210059 PMC6910861 31849480

[19] Li W, Liang J, Zhang Z, Lou H, Zhao L, Xu Y, Ou R (2017 May) MicroRNA-329-3p Targets MAPK1 to Suppress Cell Proliferation, Migration and Invasion in Cervical Cancer. Oncol Rep 37(5):2743–2750 10.3892/or.2017.5555 28393232

[20] Zhou Y, Rassy E, Coutte A, Achkar S, Espenel S, Genestie C, Pautier P, Morice P, Gouy S, Chargari C (2022) Current Standards in the Management of Early and Locally Advanced Cervical Cancer: Update on the Benefit of Neoadjuvant/Adjuvant Strategies. Cancers (Basel). May 16;14(10):2449 10.3390/cancers14102449 PMC9139662 35626051

[21] Mampre D, Mehkri Y, Rajkumar S, Sriram S, Hernandez J, Lucke-Wold B, Chandra V (2022 Jun) Treatment of breast cancer brain metastases: radiotherapy and emerging preclinical approaches. Diagnostics and Therapeutics 1(1):25–38

[22] Winkle M, El-Daly SM, Fabbri M, Calin GA (2021 Aug) Noncoding RNA therapeutics - challenges and potential solutions. Nat Rev Drug Discov 20(8):629–651 10.1038/s41573-021-00219-z PMC8212082 34145432

[23] Tim R, Mercer 1 ME, Dinger JS, Mattick (2009 Mar) Long Non-Coding RNAs: Insights Into Functions. Nat Rev Genet 10(3):155–159 10.1038/nrg2521 19188922

[24] Ke Su Q, Zhao A, Bian C, Wang Y, Cai Y, Zhang A novel positive feedback regulation between long noncoding RNA UICC and IL-6/STAT3 signaling promotes cervical cancer progression. Am J Cancer Res. 2018 Jul 1;8(7):1176–1189 PMC6079148 30094092

[25] Hu YC, Wang AM, Lu JK, Cen R, Liu LL (2017 Nov) Long noncoding RNA HOXD-AS1 regulates proliferation of cervical cancer cells by activating Ras/ERK signaling pathway. Eur Rev Med Pharmacol Sci 21(22):5049–5055 10.26355/eurrev_201711_13817 29228418

[26] Zhao X, Liu Y, Li Z, Zheng S, Wang Z, Li W, Bi Z, Li L, Jiang Y, Luo Y, Lin Q, Fu Z (2018 Jan) Chen Rufu. Linc00511 Acts as a Competing Endogenous RNA to Regulate VEGFA Expression Through Sponging hsa-miR-29b-3p in Pancreatic Ductal Adenocarcinoma. J Cell Mol Med 22(1):655–667 10.1111/jcmm.13351 PMC5742682 28984028

[27] Yu CL, Xu XL, Yuan F (2019 Sep) LINC00511 is associated with the malignant status and promotes cell proliferation and motility in cervical cancer. Biosci Rep 13(9):BSR20190903 10.1042/BSR20190903 PMC6744586 31434692

[28] Li Zhu H, Tu Y, Liang D, Tang (2018) MiR-218 Produces Anti-Tumor Effects on Cervical Cancer Cells in Vitro. World J Surg Oncol. Oct 12;16(1):204 10.1186/s12957-018-1506-3 PMC6186038 30314496

[29] Sanches JGislaineP, Xu Y, Yabasin IB, Li M, Lu Y, Xiu X, Wang Lu, Mao L, Shen J, Wang B, Hou L, Ju J, Zhao J (2018 Apr) miR-501 Is Upregulated in Cervical Cancer and Promotes Cell Proliferation, Migration and Invasion by Targeting CYLD. Chem Biol Interact 1:285:85–95 10.1016/j.cbi.2018.02.024 29477382

[30] Xu G-S, Li Z-W, Huang Z-P, Brunicardi FC, Jia Fu, Song C, Zou H-J, Sun R-F (2019 Oct) MiR-497-5p Inhibits Cell Proliferation and Metastasis in Hepatocellular Carcinoma by Targeting Insulin-Like Growth Factor 1. Mol Genet Genomic Med 7(10):e00860 10.1002/mgg3.860 PMC6785451 31441605

[31] Huang X, Wang L, Liu W, Li F (2019 Mar) MicroRNA-497-5p Inhibits Proliferation and Invasion of Non-Small Cell Lung Cancer by Regulating FGF2. Oncol Lett 17(3):3425–3431 10.3892/ol.2019.9954 PMC6396182 30867780

[32] Sun C-B, Wang H-Y, Han X-Q, Liu Y-N, Wang M-C, Zhang H-X, Gu Y-F (2020 Apr) Xiao-Gang Leng. LINC00511 Promotes Gastric Cancer Cell Growth by Acting as a ceRNA. World J Gastrointest Oncol 15(4):394–404 10.4251/wjgo.v12.i4.394 PMC7191338 32368318

[33] Wen Guo Q, Yu M, Zhang F, Li Yu, Liu W, Jiang H, Jiang H, Li (2019) Long Intergenic Non-Protein Coding RNA 511 Promotes the Progression of Osteosarcoma Cells Through Sponging microRNA 618 to Upregulate the Expression of Maelstrom. Aging (Albany NY). 6:5351–536715 10.18632/aging.102109 PMC6710040 31386627

[34] Sun Y, Liu WZ, Liu T, Feng X, Yang N, Zhou HF (2015). Signaling pathway of MAPK/ERK in cell proliferation, differentiation, migration, senescence and apoptosis. J Recept Signal Transduct Res.

[35] Owaki H, Makar R, Boulton TG, Cobb MH, Geppert TD (1992) Extracellular signal-regulated kinases in T cells: characterization of human ERK1 and ERK2 cDNAs. Biochem Biophys Res Commun. Feb 14;182(3):1416-22 10.1016/0006-291x(92)91891-s 1540184

[36] Dinesh Upadhya M, Ogata, Lixing W, Reneker (2013 Apr) MAPK1 is required for establishing the pattern of cell proliferation and for cell survival during lens development. Development 140(7):1573–1582 10.1242/dev.081042 PMC3596996 23482492

[37] Masanobu Tsubaki T, Takeda N, Ogawa K, Sakamoto H, Shimaoka A, Fujita T, Itoh M, Imano T, Ishizaka (2015 Apr) Takao Satou, Shozo Nishida. Overexpression of survivin via activation of ERK1/2, Akt, and NF-κB plays a central role in vincristine resistance in multiple myeloma cells. Leuk Res 39(4):445–452 10.1016/j.leukres.2015.01.016 25726084

[38] You B, Yang Y-L, Xu Z, Dai Y, Liu S, Mao J-H, Tetsu O, Li H, Jablons DM (2015 Feb) Liang You. Inhibition of ERK1/2 down-regulates the Hippo/YAP signaling pathway in human NSCLC cells. Oncotarget 28(6):4357–4368 10.18632/oncotarget.2974 PMC4414195 25738359

[39] Li X-W, Tuergan M, Abulizi G (2015 Nov) Expression of MAPK1 in cervical cancer and effect of MAPK1 gene silencing on epithelial-mesenchymal transition, invasion and metastasis. Asian Pac J Trop Med 8(11):937–943 10.1016/j.apjtm.2015.10.004 26614994

